# PCOS and Inositols: Controversial Results and Necessary Clarifications. Basic Differences Between D-Chiro and Myo-Inositol

**DOI:** 10.3389/fendo.2021.660381

**Published:** 2021-04-06

**Authors:** Giovanni Monastra, Ivana Vucenik, Abdel Halim Harrath, Saleh H. Alwasel, Zdravko A. Kamenov, Antonio Simone Laganà, Noemi Monti, Valeria Fedeli, Mariano Bizzarri

**Affiliations:** ^1^ Systems Biology Group Lab (SBGLab), Sapienza University, Rome, Italy; ^2^ Department of Medical and Research Technology, University of Maryland School of Medicine, Baltimore, MD, United States; ^3^ Department of Zoology, College of Science, King Saud University, Riyadh, Saudi Arabia; ^4^ Department of Internal Medicine, Medical University of Sofia, Sofia, Bulgaria; ^5^ Department of Obstetrics and Gynecology, “Filippo Del Ponte” Hospital, University of Insubria, Varese, Italy; ^6^ Department of Experimental Medicine, Systems Biology Group Lab, University Sapienza, Rome, Italy

**Keywords:** PCOS, myo-inositol, D-chiro inositol, metabolism, ovarian cells

## A New Approaches in PCOS Treatment

Myo-Inositol (myo-Ins) and its phosphate derivatives—including inositol phosphates (InsPs), inositol pyrophosphates (IPPs) and phosphatidyl-inositol phosphate (PtdIns)—are credited to act as second messengers, which accumulate rapidly and transiently in response to external or endocrine signals, a phenomenon that allows signaling to be discrete and regulated (1, 2). Noticeably, inositol is involved in the transduction of several endocrine signals, including insulin (3, 4), thyroid hormones (5), gonadotropins (6), lipids with hormone-like activity (as prostaglandins) (7), and many other endocrine systems (8). Namely, in the last decade, a growing body of clinical and experimental research provided robust evidence about the efficiency of inositol in reversing a few clinical, metabolic, and endocrine features of the Polycystic Ovary Syndrome (PCOS). Myo-inositol, alone or in combination with its isomer D-Chiro-Inositol (D-Chiro-Ins), showed to exert a variable—albeit significant—effect in improving both symptoms and outcome in PCOS patients (9). Experimental and pilot clinical studies pointed out that a combination of both isomers could provide a reliable rationale for establishing a proper treatment strategy, as first suggested by Beemster’s seminal study (10, 11).

However, the proper formula—i.e., the respective percentage of myo-Ins and D-Chiro-Ins—is still a matter of debate. In several cases, no conclusive insights can be obtained from clinical trials based on unclear rational design, limited number of recruited patients and variable formula composition and dosage(s).

First, it is improper to compare clinical results from studies in which commercial nutraceutical formulas involve a wide range of concentrations (**Table 1**), with the myo-Ins/D-Chiro-Ins ratio varying implausibly from 0.4:1 to 104:1.

**Table 1:** Different myo-Ins/D-chiro-Ins ratios in commercial nutraceutical formulas.

N.	myo-Ins/D-chiro-Ins	Quantities of myo-Ins and D-chiro-Ins
1	0.4:1	200 mg myo-Ins + 500 mg D-chiro-Ins
2	0.8:1	200 mg myo-Ins + 250 mg D-chiro-Ins
3	3.6:1	1.100 mg myo-Ins + 300 mg D-chiro-Ins
4	4:1	2 g myo-Ins + 500 mg D-chiro-Ins
5	5:1	1 g myo-Ins + 200 mg D-chiro-Ins
6	7:1	875 mg myo-Ins + 125 mg D-chiro-Ins
7	8:1	2 g myo-Ins + 250 mg D-chiro-Ins
8	9.5:1	2 g myo-Ins + 210.5 mg D-chiro-Ins
9	10:1	480 mg myo-Ins + 43 mg D-chiro-Ins
10	40:1	2 g myo-Ins + 50 mg D-chiro-Ins
11	66:1	2 g myo-Ins + 30 mg D-chiro-Ins
12	92:1	2.03 g myo-Ins + 0.02g D-chiro-Ins
13	104:1	1,980 mg myo-Ins + 19 mg D-chiro-Ins

Current commercial preparations also contain D-Chiro-Ins alone at concentrations reaching 600 mg that can be administered once or twice a day. Therefore, the daily dose of D-chiro-Ins, alone or with myo-Ins, ranges from low (less than 300 mg/die), medium (300–600 mg/die) and high (600–1,200 mg/die).

## Inositols as Endocrine Modulators

No plausible hypothesis can justify these bizarre “compositions”, and consequently is not surprisingly that so different results have been gathered with nutraceutical supports deprived of any scientific evidence. We would also ask if it is ethical to permit that such “drugs”, while deceiving consumers, should invade the market, baffling both the Regulatory Agency’s control, as well as common sense. It is also regrettable that in some cases scientific journals have not felt the duty to inform correctly, by underestimating potential conflict of interest and biased methodological approaches (12), thus contributing to worsen the “epidemic” of controversial/irreproducible/biased results that plague current clinical research (13).

Second, several published reports (14) seems to ignore that myo-Ins and D-Chiro-Ins display opposite effects upon ovary and steroidogenesis, despite both improve insulin transduction and glucose utilization, through their inositolphosphoglycans (IPGs) derivatives (15). However important it may be, imbalance in glucose and insulin metabolism cannot explain by itself the overall history of PCOS pathogenesis.

Indeed, some evidence suggests that D-Chiro-Ins can directly regulate steroidogenic enzyme genes in human granulosa cells, reducing the mRNA expression of both aromatase and cytochrome P450 side-chain cleavage genes in a dose–response fashion (16). Furthermore, D-Chiro-Ins increases testosterone levels in theca cells from PCOS women (17). Those data should have suggested caution in treating PCOS women with high doses of D-Chiro-Ins. Indeed, despite the promising results obtained in a pivotal study (18), a subsequent investigation using high D-Chiro-Ins dosages was unable to confirm those preliminary results and was discontinued (19).

On the other hand, myo-Ins participates in the modulation of the FSH signaling, as suggested by clinical and experimental data. Indeed, upon myo-Ins treatment, LH/FSH ratio in plasma of PCOS women significantly decreases (20–22), while *in vitro* fertilization myo-Ins supplementation allows to significantly reduce the amount of recombinant FSH administered (6, 23). In turn, FSH stimulates aromatase synthesis, a critical step in androgen conversion to estrogens and in terminal follicle maturation (24). It is worth noting that downregulation of FSH and subsequent dramatic decrease in aromatase synthetized by granulosa cells constitute a hallmark of PCOS (25). Noticeably, while D-Chiro-Ins inhibits aromatase as previously recalled, myo-Ins seems to enhance aromatase synthesis in granulosa as well as in endocrine-responsive breast cancer, probably acting also through a direct genomic modulation, given that in breast cancer cell the observed increase in aromatase is independent from FSH stimulation (26). Moreover, myo-Ins can modulate steroidogenesis in the ovary by exerting complex effects upon cytoskeleton architecture (27). Therefore, it is not surprising that in physiological homeostatic conditions, the myo-Ins/D-Chiro-Ins ratio in ovarian tissues is kept within the range of 70–00:1, while in ovaries from PCOS women this ratio is pathologically decreased (28, 29). Conversely, high levels of D-Chiro-Ins show detrimental effects upon blastocyst quality (30). An appreciable step forward in understanding the tricky interplay in between myo-Ins and D-Chiro-Ins in modulating the ovarian physiology has been carried out by Bevilacqua et al. in a recent paper in which the effectiveness of different myo-Ins/D-Chiro-Ins formulas was experimentally addressed in PCOS mice (31). This study provided the first experimental evidence of the different efficacy exerted by various myo-Ins/D-Chiro-Ins ratios (5:1; 20:1; 40:1; 80:1) in restoring a normal phenotype. Mice treated daily with 420 mg/kg myo-Ins/D-Chiro-Ins in a 40:1 molar ratio made a fast and almost full recovery from PCOS signs and symptoms. On the contrary, the other myo-Ins/D-Chiro-Ins ratios were less effective or had even negative effects. In particular, the formulation with higher D-Chiro-Ins content proved to worsen the PCOS pathological features. Some clinical studies have recently confirmed these results in PCOS women (32), thus vindicating the keen insight first advanced by Unfer (33, 34).

Furthermore, administration of high concentrations of D-Chiro-Ins can have not only detrimental effects upon follicle functions but can also impair myo-Ins availability. A recent study by Garzon et al. showed that 1 g D-Chiro-Ins significantly reduces the intestinal absorption of 6 g myo-Ins (35). The same effect can be obtained by administering maltodextrin, sorbitol (36) or phlorizin (37), a non-transported competitive inhibitor of sodium-coupled sugar co-transporters. In the study by Garzon et al. (35), performed in female and male volunteers, the plasma myo-Ins/D-Chiro-Ins ratio was 6:1, which is strongly in favor of D-Chiro-Ins, when compared to the physiological value (40:1) (38).

This effect can be explained by hypothesizing a competitive action upon inositol transporter. In fact, SMIT2 transports myo-Ins with an average K_M_ of 120–150 μM, with good agreement with plasma levels of myo-Ins (32.5 ± 1.5 μM, ranging from 26.8 to 43.0 μM) (39). D-Chiro-Ins is transported with an average K_M_ of 110–130 μM, similar to myo-Ins; nevertheless, the average plasma concentration of D-Chiro-Ins is usually less than 100 nM and hence it is unlikely that it can interfere with myo-Ins absorption under normal conditions (40). Moreover, this observation implies that in the physiological setting SMIT2-based transport is only marginally committed in ensuring D-Chiro-Ins absorption, and D-Chiro-Ins could hardly impair myo-Ins uptake. However, when D-Chiro-Ins is administered at high dosage (i.e., ≥1 g), it could efficiently compete with myo-Ins for absorption at the gut level, thus decreasing the myo-Ins/D-Chiro-Ins ratio in plasma. Conversely, while pharmaceutical formulas in the physiological ratio of 40:1 are unlikely to impair myo-Ins uptake, other studies in which high D-Chiro-ins concentrations were used can significantly reduce myo-Ins bioavailability.

The unexpected competitive inhibition exerted by D-Chiro-Ins as well by other small sugar-like molecules may help explaining the phenomenon of the so-called “inositol resistance”—highlighted by several clinical trials—that could account for 30–40% of inositol failure in PCOS treatment (41). Resistance can be ascribed to insufficient inositol availability, as suggested by studies in which the intestinal absorption of inositols significantly increases in humans upon co-administration of α-lactalbumin that reversibly opens the tight junctions (42). Noticeably, the association of myo-Ins with α-lactalbumin demonstrated to be beneficial in restoring ovulation and in improving PCOS features by counteracting “inositol resistance” in a group of PCOS women (43). This study confirms how relevant could be achieving high plasma levels of myo-Ins in the therapeutic management of PCOS.

At the very beginning, studies on inositol in PCOS management were mostly influenced by metabolic considerations, given that D-Chiro-Ins is required to allow insulin transduction (44) It should be recalled that myo-Ins epimerization into D-Chiro-Ins is under the control of insulin, which tightly modulate the conversion rate according to the tissues needs (45). Noteworthy, both insulin resistance and diabetes type 2 have been associated with reduced availability of D-Chiro-Ins, suggesting that it should act as insulin second messenger and insulin-sensitizing agent (46).

The highest conversion rate of myo-Ins into D-Chiro-Ins is close to 9%, and was detected in liver and muscle, two important insulin sensitive areas, whereas in hearth and brain is less than 2% (47). Remarkably, in biological fluids the myo-Ins/D-Chiro-Ins ratio ranges from 40:1, in plasma, to 100:1 in follicular fluid (28). Differences in in the ratio values highlight that tissues display significant differential needs of both isomers. Insulin resistance can dramatically impair D-Chiro-Ins levels in many tissues, with resulting low intracellular levels of D-Chiro-Ins (48). Conversely, restoring D-Chiro-Ins levels proved to be beneficial in ameliorating diabetes-related metabolic features, and in reducing the insulin stimulation upon theca cells and, hence, the consequent insulin-dependent enhancement of androgenesis (49), given that insulin-dependent stimulation of ovarian epimerase will be appreciably tempered upon D-Chiro-Ins supplementation. However, D-Chiro-Ins administration exceeding the need to mitigate insulin secretion would likely impair ovary function by dramatically decreasing myo-Ins levels in the ovary, as demonstrated by Larner’s seminal paper (29). However, this “metabolic” model is biased by the assumption that PCOS pathogenesis mostly relies upon a metabolic “defect” (i.e., defective insulin transduction due to impaired availability of inositolphosphoglycans) and underestimates the steroidogenic effects directly triggered by myo-Ins and especially by D-Chiro-Ins. Exceeding doses of D-Chiro-Ins may indeed enhance androgenesis and hamper aromatase synthesis, as previously mentioned. Probably, the role of insulin resistance in underpinning PCOS pathogenesis has been too much emphasized since the first report in which a link was hypothesized in between PCOS and defects in insulin/glucose metabolism (50). Yet, the whole evidence related to D-Chiro-Ins is still scarce and should be urgently updated through focused investigations, namely by ascertaining the different evolution of PCOS associated with mixed insulin resistance compared to PCOS with exclusively muscular or exclusively hepatic insulin resistance (51).

## The Right Formula

Overall, those data suggest that the choice of the respective concentrations of the two isomers should be carefully weighted and not left to improvisation. Namely, if the aim pursued is that of improving the ovary responsiveness to the FSH-Aromatase endocrine axis—as is the case in PCOS management—high dosages of D-Chiro-Ins should be avoided. After all, the fact that D-Chiro-Ins is usually excreted in urine in large amounts, showing a higher urinary D-Chiro-Ins/myo-Ins ratio, seems to suggest that kidney can selectively concentrates and then excretes D-Chiro-Ins in excess, which can be potentially harmful (47). Different D-Chiro-Ins dosages can be eventually conceived (and properly tested) in specific pathological conditions (anti-estrogenic treatments, diabetes)?, with the aim to primarily inhibit aromatase activity or foster insulin activity in non-ovarian tissues (52). However, it should be borne in mind that high dosages of D-Chiro-Ins should be avoided in PCOS patients. Instead, their administration can be useful if the therapeutic goal is to reduce aromatase activity and/or increase testosterone levels.

It is increasingly appreciated that PCOS may assume different clinical phenotypes and consequently proper assessment of the biochemical and medical features of each patho-phenotypes are required to adopt the best effective therapeutic strategy (53). Accordingly, it can be surmised that inositol-based treatments should be tailored for those specific clinical PCOS phenotypes for which a robust evidence has been provided.

Thereby, future studies are mandatory to ascertain the molecular basis of inositol activity upon ovarian cells and to investigate the beneficial effects of an appropriate myo-Ins/D-Chiro-Ins formula on larger cohorts of patients and on different PCOS phenotypes, besides promising results have been hitherto obtained with pharmacological formulas in which the myo-Ins/D-Chiro-Ins ratio is set at 40:1, according to the physiological plasma value (54). For now, empirical solutions, based on inappropriate pathogenic models and baseless statements, should be mandatorily avoided.

## Author Contributions

GM, MB, IV, ZK, AH, SA and AL: conceptualization and writing. AH and SA: funding acquisition. NM and VF: supervision, revision and editing. All authors contributed to the article and approved the submitted version.

## Funding

AH, SA, and MB extend their appreciation to the International Scientific Partnership program ISPP at King Saud University for funding this research work through ISPP-122.

## Conflict of Interest

In the past, GM was employed as advisor by the company Lo.Li Pharma Co., Italy.

The authors declare that this study did not receive any funding from private companies. Supporting funds were only received from King Saud University. The funder was not involved in the study design, collection, analysis, interpretation of data, the writing of this article or the decision to submit it for publication.

The remaining authors declare that the research was conducted in the absence of any commercial or financial relationships that could be construed as a potential conflict of interest.

## References

[1] Hughes AR Horstman DA Takemura H Putney JW Jr. Inositol phosphate metabolism and signal transduction. Am Rev Respir Dis (1990) 141(3 Pt 2):S115–8. 10.1164/ajrccm/141.3_Pt_2.S115 2155557

[2] Chakraborty A Kim S Snyder SH. Inositol pyrophosphates as mammalian cell signals. Sci Signal (2011) 4(188):re1. 10.1126/scisignal.2001958 21878680 PMC3667551

[3] Jones DR Varela-Nieto I. Diabetes and the role of inositol-containing lipids in insulin signaling. Mol Med (1999) 5(8):505–14. 10.1007/BF03401978 PMC2230454 10501653

[4] Larner J Brautigan DL Thorner MO. D-chiro-inositol glycans in insulin signaling and insulin resistance. Mol Med (2010) 16(11-12):543–52. 10.2119/molmed.2010.00107 PMC2972396 20811656

[5] Benvenga S Antonelli A. Inositol(s) in thyroid function, growth and autoimmunity. Rev Endocr Metab Disord (2016) 17(4):471–84. 10.1007/s11154-016-9370-3 27315814

[6] Lagana AS Vitagliano A Noventa M Ambrosini G D’Anna R. Myo-inositol supplementation reduces the amount of gonadotropins and length of ovarian stimulation in women undergoing IVF: a systematic review and meta-analysis of randomized controlled trials. Arch Gynecol Obstet (2018) 298(4):675–84. 10.1007/s00404-018-4861-y 30078122

[7] Sales KJ Milne SA Williams AR Anderson RA Jabbour HN. Expression, localization, and signaling of prostaglandin F2 alpha receptor in human endometrial adenocarcinoma: regulation of proliferation by activation of the epidermal growth factor receptor and mitogen-activated protein kinase signaling pathways. J Clin Endocrinol Metab (2004) 89(2):986–93. 10.1210/jc.2003-031434 14764825

[8] Catt KJ Hunyady L Balla T. Second messengers derived from inositol lipids. J Bioenerg Biomembr (1991) 23(1):7–27. 10.1007/BF00768836 2010435

[9] Lagana AS Garzon S Casarin J Franchi M Ghezzi F. Inositol in Polycystic Ovary Syndrome: Restoring Fertility through a Pathophysiology-Based Approach. Trends Endocrinol Metab (2018) 29(11):768–80. 10.1016/j.tem.2018.09.001 30270194

[10] Beemster P Groenen P Steegers-Theunissen R. Involvement of inositol in reproduction. Nutr Rev (2002) 60(3):80–7. 10.1301/00296640260042748 11908744

[11] Dinicola S Chiu TT Unfer V Carlomagno G Bizzarri M. The rationale of the myo-inositol and D-chiro-inositol combined treatment for polycystic ovary syndrome. J Clin Pharmacol (2014) 54(10):1079–92. 10.1002/jcph.362 25042908

[12] Laganà A Aragona C. Effects of oral supplementation with Inositols in women with polycystic ovary syndrome undergoing in vitro fertilization: a long and windy road. Organisms (2019) 3(1):11–3. 10.13133/2532-5876_5.3

[13] Ioannidis JP. Why most published research findings are false. PloS Med (2005) 2(8):e124. 10.1371/journal.pmed.0020124 16060722 PMC1182327

[14] Genazzani A. Inositols: reflections on how to choose the appropriate one for PCOS. Gynecol Endocrinol (2020) 36(12):1045–6. 10.1080/09513590.2020.1846697 33169639

[15] Paul C Lagana AS Maniglio P Triolo O Brady DM. Inositol’s and other nutraceuticals’ synergistic actions counteract insulin resistance in polycystic ovarian syndrome and metabolic syndrome: state-of-the-art and future perspectives. Gynecol Endocrinol (2016) 32(6):431–8. 10.3109/09513590.2016.1144741 26927948

[16] Sacchi S Marinaro F Tondelli D Lui J Xella S Marsella T. Modulation of gonadotrophin induced steroidogenic enzymes in granulosa cells by d-chiroinositol. Reprod Biol Endocrinol (2016) 14(1):52. 10.1186/s12958-016-0189-2 27582109 PMC5006365

[17] Nestler JE Jakubowicz DJ de Vargas AF Brik C Quintero N Medina F. Insulin stimulates testosterone biosynthesis by human thecal cells from women with polycystic ovary syndrome by activating its own receptor and using inositolglycan mediators as the signal transduction system. J Clin Endocrinol Metab (1998) 83(6):2001–5. 10.1210/jcem.83.6.4886 9626131

[18] Nestler JE Jakubowicz DJ Reamer P Gunn RD Allan G. Ovulatory and metabolic effects of D-chiro-inositol in the polycystic ovary syndrome. N Engl J Med (1999) 340(17):1314–20. 10.1056/NEJM199904293401703 10219066

[19] Cheang KI Baillargeon JP Essah PA Ostlund RE Jr Apridonize T Islam L. Insulin-stimulated release of D-chiro-inositol-containing inositolphosphoglycan mediator correlates with insulin sensitivity in women with polycystic ovary syndrome. Metabolism (2008) 57(10):1390–7. 10.1016/j.metabol.2008.05.008 PMC2574418 18803944

[20] Pizzo A Lagana AS Barbaro L. Comparison between effects of myo-inositol and D-chiro-inositol on ovarian function and metabolic factors in women with PCOS. Gynecol Endocrinol (2014) 30(3):205–8. 10.3109/09513590.2013.860120 24351072

[21] Artini PG Di Berardino OM Papini F Genazzani AD Simi G Ruggiero M. Endocrine and clinical effects of myo-inositol administration in polycystic ovary syndrome. A randomized study. Gynecol Endocrinol (2013) 29(4):375–9. 10.3109/09513590.2012.743020 23336594

[22] Costantino D Minozzi G Minozzi E Guaraldi C. Metabolic and hormonal effects of myo-inositol in women with polycystic ovary syndrome: a double-blind trial. Eur Rev Med Pharmacol Sci (2009) 13(2):105–10. 19499845

[23] Emekci Ozay O Ozay AC Cagliyan E Okyay RE Gulekli B. Myo-inositol administration positively effects ovulation induction and intrauterine insemination in patients with polycystic ovary syndrome: a prospective, controlled, randomized trial. Gynecol Endocrinol (2017) 33(7):524–8. 10.1080/09513590.2017.1296127 28277112

[24] Stocco C. Tissue physiology and pathology of aromatase. Steroids. (2012) 77(1-2):27–35. 10.1016/j.steroids.2011.10.013 22108547 PMC3286233

[25] Erickson GF Hsueh AJ Quigley ME Rebar RW Yen SS. Functional studies of aromatase activity in human granulosa cells from normal and polycystic ovaries. J Clin Endocrinol Metab (1979) 49(4):514–9. 10.1210/jcem-49-4-514 479344

[26] Gambioli R Forte G Aragona C Bevilacqua A Bizzarri M Unfer V. The use of D-chiro-Inositol in clinical practice. Eur Rev Med Pharmacol Sci (2021) 25(1):438–46. 10.26355/eurrev_202101_24412 33506934

[27] Bizzarri M Cucina A Dinicola S Harrath AH Alwasel SH Unfer V. Does myo-inositol effect on PCOS follicles involve cytoskeleton regulation? Med Hypotheses (2016) 91:1–5. 10.1016/j.mehy.2016.03.014 27142131

[28] Unfer V Carlomagno G Papaleo E Vailati S Candiani M Baillargeon JP. Hyperinsulinemia Alters Myoinositol to d-chiroinositol Ratio in the Follicular Fluid of Patients With PCOS. Reprod Sci (2014) 21(7):854–8. 10.1177/1933719113518985 24501149

[29] Heimark D McAllister J Larner J. Decreased myo-inositol to chiro-inositol (M/C) ratios and increased M/C epimerase activity in PCOS theca cells demonstrate increased insulin sensitivity compared to controls. Endocr J (2014) 61(2):111–7. 10.1507/endocrj.EJ13-0423 24189751

[30] Ravanos K Monastra G Pavlidou T Goudakou M Prapas N. Can high levels of D-chiro-inositol in follicular fluid exert detrimental effects on blastocyst quality? Eur Rev Med Pharmacol Sci (2017) 21(23):5491–8. 10.26355/eurrev_201712_13940 29243796

[31] Bevilacqua A Dragotto J Giuliani A Bizzarri M. Myo-inositol and D-chiro-inositol (40:1) reverse histological and functional features of polycystic ovary syndrome in a mouse model. J Cell Physiol (2019) 234(6):9387–98. 10.1002/jcp.27623 30317628

[32] Nordio M Basciani S Camajani E. The 40:1 myo-inositol/D-chiro-inositol plasma ratio is able to restore ovulation in PCOS patients: comparison with other ratios. Eur Rev Med Pharmacol Sci (2019) 23(12):5512–21. 10.26355/eurrev_201906_18223 31298405

[33] Carlomagno G Unfer V Roseff S. The D-chiro-inositol paradox in the ovary. Fertil Steril (2011) 95(8):2515–6. 10.1016/j.fertnstert.2011.05.027 21641593

[34] Nestler JE Unfer V. Reflections on inositol(s) for PCOS therapy: steps toward success. Gynecol Endocrinol (2015) 31(7):501–5. 10.3109/09513590.2015.1054802 26177098

[35] Garzon S Lagana AS Monastra G. Risk of reduced intestinal absorption of myo-inositol caused by D-chiro-inositol or by glucose transporter inhibitors. Expert Opin Drug Metab Toxicol (2019) 15(9):697–703. 10.1080/17425255.2019.1651839 31382802

[36] Cammarata PR Chen HQ Yang J Yorio T. Modulation of myo-[3H]inositol uptake by glucose and sorbitol in cultured bovine lens epithelial cells. II. Characterization of high- and low-affinity myo-inositol transport sites. Invest Ophthalmol Vis Sci (1992) 33(13):3572–80. 1464503

[37] Coady MJ Wallendorff B Gagnon DG Lapointe JY. Identification of a novel Na+/myo-inositol cotransporter. J Biol Chem (2002) 277(38):35219–24. 10.1074/jbc.M204321200 12133831

[38] Facchinetti F Giulia D Neri I. The Ratio of MI to DCI and Its Impact in the Treatment of Polycystic Ovary Syndrome: Experimental and Literature Evidences. Front Gynecol Endocrinol (2016) 3:103–9. 10.1007/978-3-319-23865-4_13

[39] Leung KY Mills K Burren KA Copp AJ Greene ND. Quantitative analysis of myo-inositol in urine, blood and nutritional supplements by high-performance liquid chromatography tandem mass spectrometry. J Chromatogr B Analyt Technol BioMed Life Sci (2011) 879(26):2759–63. 10.1016/j.jchromb.2011.07.043 PMC3632838 21856255

[40] Ostlund RE Jr McGill JB Herskowitz I Kipnis DM Santiago JV. Sherman WR. D-chiro-inositol metabolism in diabetes mellitus. Proc Natl Acad Sci U.S.A. (1993) 90(21):9988–92. 10.1073/pnas.90.21.9988 PMC47698 8234346

[41] Kamenov Z Kolarov G Gateva A Carlomagno G Genazzani AD. Ovulation induction with myo-inositol alone and in combination with clomiphene citrate in polycystic ovarian syndrome patients with insulin resistance. Gynecol Endocrinol (2015) 31(2):131–5. 10.3109/09513590.2014.964640 25259724

[42] Monastra G Sambuy Y Ferruzza S Ferrari D Ranaldi G. Alpha-lactalbumin Effect on Myo-inositol Intestinal Absorption: In vivo and In vitro. Curr Drug Deliv (2018) 15(9):1305–11. 10.2174/1567201815666180509102641 29745333

[43] Montanino Oliva M Buonomo G Calcagno M Unfer V. Effects of myo-inositol plus alpha-lactalbumin in myo-inositol-resistant PCOS women. J Ovarian Res (2018) 11(1):38. 10.1186/s13048-018-0411-2 29747700 PMC5944130

[44] Larner J Huang LC Schwartz CF Oswald AS Shen TY Kinter M. Rat liver insulin mediator which stimulates pyruvate dehydrogenase phosphate contains galactosamine and D-chiroinositol. Biochem Biophys Res Commun (1988) 151(3):1416–26. 10.1016/S0006-291X(88)80520-5 2833261

[45] Bizzarri M Fuso A Dinicola S Cucina A Bevilacqua A. Pharmacodynamics and pharmacokinetics of inositol(s) in health and disease. Expert Opin Drug Metab Toxicol (2016) 12(10):1181–96. 10.1080/17425255.2016.1206887 27351907

[46] Larner J. D-chiro-inositol–its functional role in insulin action and its deficit in insulin resistance. Int J Exp Diabetes Res (2002) 3(1):47–60. 10.1080/15604280212528 11900279 PMC2478565

[47] Pak Y Huang LC Lilley KJ Larner J. In vivo conversion of [3H]myoinositol to [3H]chiroinositol in rat tissues. J Biol Chem (1992) 267(24):16904–10. 10.1016/S0021-9258(18)41870-4 1387397

[48] Asplin I Galasko G Larner J. chiro-inositol deficiency and insulin resistance: a comparison of the chiro-inositol- and the myo-inositol-containing insulin mediators isolated from urine, hemodialysate, and muscle of control and type II diabetic subjects. Proc Natl Acad Sci USA (1993) 90(13):5924–8. 10.1073/pnas.90.13.5924 PMC46839 8392181

[49] Baptiste CG Battista MC Trottier A Baillargeon JP. Insulin and hyperandrogenism in women with polycystic ovary syndrome. J Steroid Biochem Mol Biol (2010) 122(1-3):42–52. 10.1016/j.jsbmb.2009.12.010 20036327 PMC3846536

[50] Larner J Craig JW. Urinary myo-inositol-to-chiro-inositol ratios and insulin resistance. Diabetes Care (1996) 19(1):76–8. 10.2337/diacare.19.1.76 8720541

[51] Yilmaz M Bukan N Ersoy R Karakoç A Yetkin I Ayvaz G. Glucose intolerance, insulin resistance and cardiovascular risk factors in first degree relatives of women with polycystic ovary syndrome. Hum Reprod (2005) 20:2414–20. 10.1093/humrep/dei070 15890734

[52] Lagana AS Garzon S Unfer V. New clinical targets of d-chiro-inositol: rationale and potential applications. Expert Opin Drug Metab Toxicol (2020) 16(8):703–10. 10.1080/17425255.2020.1785429 32552009

[53] Aversa A La Vignera S Rago R Gambineri A Nappi RE Calogero AE. Fundamental Concepts and Novel Aspects of Polycystic Ovarian Syndrome: Expert Consensus Resolutions. Front Endocrinol (Lausanne) (2020) 11:516. 10.3389/fendo.2020.00516 32849300 PMC7431619

[54] Facchinetti F Unfer V Dewailly D Kamenov ZA Diamanti-Kandarakis E Lagana AS. Inositols in Polycystic Ovary Syndrome: An Overview on the Advances. Trends Endocrinol Metab (2020) 31(6):435–47. 10.1016/j.tem.2020.02.002 32396844

